# Enhanced Rehydration of Micellar Casein Powder: Effects of Electrodialysis Treatment

**DOI:** 10.3390/foods14244171

**Published:** 2025-12-05

**Authors:** Kerong Wang, Yun Chen, Xuhui Fan, Shengbo Yu, Yang Song, Shuang Wang, Weibo Zhang, Pengjie Wang, Shumin Wang, Yanli Zhu, Chong Chen, Zhishen Mu

**Affiliations:** 1 Department of Nutrition and Health, China Agricultural University, Beijing 100193, China; 15534892026@163.com (K.W.); 15385662159@163.com (X.F.); sy20243313941@cau.edu.cn (Y.S.); wangshuang@cau.edu.cn (S.W.); zhangweibo@cau.edu.cn (W.Z.); wpj1019@cau.edu.cn (P.W.); 2 National Enterprise Technology Center, Inner Mongolia Mengniu Dairy (Group) Co., Ltd., Hohhot 011500, China; chenyun@mengniu.cn (Y.C.); yushengbo@mengniu.cn (S.Y.); 3 Global R&D Innovation Center, Inner Mongolia Mengniu Dairy (Group) Co., Ltd., Hohhot 011500, China; 4 College of Food and Bio-Engineering, Beijing Vocational College of Agriculture, Beijing 102442, China; sdutshumin@163.com; 5 School of Life Science and Engineering, Northwest Minzu University, Lanzhou 730030, China; zyanli0129@163.com

**Keywords:** casein micelle powder, electrodialysis, rehydration

## Abstract

The poor rehydration capacity of micellar casein (MC) powder due to the tightly cross-linked structure formed by colloidal calcium phosphate (CCP) limits its potential applications. This study aimed to improve the rehydration properties of MC powder by electrodialysis (ED) treatment. After being treated by ED for 0, 10, 30, 60, and 90 min, the casein micelle powder exhibited a reduction in calcium content (25.55 ± 0.08 g/kg to 17.47 ± 0.05 g/kg) with prolonged treatment, corresponding to the progressive dissociation of CCP bridges. Furthermore, the ED treatment significantly increased the solubility of casein micelle powder. Interestingly, after 60 min, the solubility of ED-treated micellar casein plateaued at approximately 80% within 30 min, likely due to modification on protein structure. Therefore, these results indicate that the structure of micellar casein can be modified through ED treatment, leading to improved rehydration properties.

## 1. Introduction

Micellar casein (MC) is widely utilized in the food industry for its roles in enhancing texture, improving nutritional value, and stabilizing emulsions in dairy and functional products [1,2,3,4,5]. Liquid MC is conventionally dried into powder for transportation and storage [6]. MC powder should have excellent rehydration properties for further applications. However, numerous reports have documented that MC powder has poor rehydration [7,8].

The Holt model has reported that MC is a tightly structured nanocluster complex formed by connecting colloidal calcium phosphate (CCP) [9]. This characteristic leads to poor solubility in water and limits its potential applications in various food and pharmaceutical products. Additionally, conventional MC powder is usually prepared using a spray-drying technique; however, this technique causes protein denaturation due to high temperature and results in poor solubility [10]. In contrast, freeze-drying (FD) preserves the native structure of MC and leads to better rehydration properties [8]. Thus, it is important to dissociate CCP and prevent protein denaturation during drying to improve solubility and functionality of MC.

Many approaches have been reported to dissociate CCP to obtain MC with improved solubility and functionality. Typically, chelating agents such as Ethylene Diamine Tetraacetic Acid [11] or citrate sequester calcium ions from CCP are capable of inducing micellar structure destabilization and enhancing solubility [12]. Notably, green physical technologies are increasingly receiving attention due to the increasing demands of effective food processing technologies and the clean-label trend [13]. Therefore, chemical treatment may not align with the clean-label trend. Moreover, residual chelators could affect the sensory and safety properties of food products. Therefore, to address these limitations, additive-free and environmentally friendly physical technologies for CCP dissociation are being extensively explored.

Electrodialysis (ED) is an electrochemical separation process that uses an electric field to drive ion migration through selective cation- and anion-exchange membranes, enabling the desalination of brackish water and concentration of a saline stream [14]. This technology has been widely applied across diverse fields, including water treatment (e.g., brackish water desalination, industrial wastewater purification) [15], food processing (e.g., whey demineralization, juice deacidification) [16], chemical and pharmaceutical industries (e.g., acid/base recovery, organic acid purification) [17], and energy production (e.g., reverse ED for salinity-gradient power generation) [18]. Thus, ED can be employed to partially remove calcium ions from CCP, enhancing the solubility of the MC powder. This study aimed to investigate the effects of ED treatment on the rehydration of MC powder. The dynamic rehydration behavior of MC powder was extensively characterized and discussed in terms of wetting, dispersion, and dissolution.

## 2. Materials and Methods

### 2.1. Materials

MC solution (total solids 19.4%, protein 84%, casein purity 92.6%) was provided by Mengniu Dairy (Group) Co., Ltd. (Hohhot, China). All chemical reagents used in this study were analytical grade and purchased from commercial suppliers. Absolute ethanol was obtained from Tianjin Zhiyuan Chemical Reagent Co., Ltd. (Tianjin, China). Tris–HCl buffer, Phosphate-Buffered Saline (PBS), and 8-Anilino-1-naphthalenesulfonic acid (ANS) were purchased from Shanghai Aladdin Biochemical Technology Co., Ltd. (Shanghai, China). Sodium dodecyl sulfate (SDS), Coomassie Brilliant Blue Rapid Staining Solution, and Ammonium persulfate were acquired from Beijing Lanbolide Commerce Co., Ltd. (Beijing, China). ProClin (TM)950 was purchased from Sigma-Aldrich Trading Co., Ltd. (Shanghai, China).

### 2.2. Electrodialysis Treatment

MC solution was treated with an ED device (Model BONA-ED-18, Shandong, China). The ED stack was configured with an alternating arrangement of anion-exchange and cation-exchange membranes, and the electrodes were constructed of titanium coated with ruthenium. The parameters were set at a constant voltage of 15 V, a flow rate of 4 L/h, and a temperature of 25 °C. The samples were loaded into the device and treated under different times (0, 10, 30, 60, and 90 min).

### 2.3. Particle Size and Zeta Potential Measurements

The particle size and zeta potential of ED-treated MC solution was determined after being diluted 100 times with simulated ultrafiltration fluid. The simulated ultrafiltration fluid is composed of KH_2_PO_4_ (1.58 g/L), K_3_ citrate·H_2_O (1.20 g/L), Na_3_ citrate·5H_2_O (2.12 g/L), K_2_SO_4_ (0.18 g/L), CaCl_2_·H_2_O (1.32 g/L), MgCl_2_·6H_2_O (0.65 g/L), K_2_CO_3_ (0.30 g/L), and KCl (0.60 g/L) [19] and thoroughly mixed to ensure complete dispersion. Subsequently, 1 mL of diluted sample was loaded into the sample cell of a Zetasizer Nano series high-performance nanoparticle analyzer [20], and the sample was measured at 25 °C. The particle size and zeta potential were measured three times to ensure the reliability and reproducibility of the results.

### 2.4. Preparation of MC Powder

The ED-treated MC solution was poured into a dish with a depth of 1 cm, covered with plastic wrap, and pre-frozen at −80 °C for 12 h. Then, the sample was placed in a freeze-dryer (LGJ-10, Beijing, China) and freeze-dried for 48 h to obtain MC powder.

### 2.5. Composition and Structure of MC Powder

#### 2.5.1. Calcium and Phosphorus Content

The mineral composition of MC powder was quantitatively analyzed by inductively coupled plasma optical emission spectrometry (ICP-OES, Agient ICP-OES 5110, Santa Clara, CA, USA) according to a previously published method [21]. Specifically, 0.5 g of MC powder was suspended in 50 mL of deionized water and gently vortexed (10 s) to achieve complete dispersion and then immediately centrifuged at 1000× *g* for 45 min at 25 °C. The supernatant filtrate was collected to determine the calcium and phosphorus content in the serum phase. Total calcium and phosphate contents were directly measured from the MC powder via ICP-OES.

#### 2.5.2. Sodium Dodecyl Sulfate Polyacrylamide Gel Electrophoresis (SDS-PAGE)

Firstly, the freeze-dried powder was dissolved and mixed with loading buffer; the volume ratio was 4:1, shaken well, and boiled for 5–8 min, and then cooled in an ice bath. An amount of 12% separating gel and 6% stacking gel were prepared, and 5 μL of the sample was loaded for both the marker and samples [22]. Then, electrophoresis was carried out in a two-step constant voltage procedure: 80 V for 30 min and 120 V for 1 h after the samples entered the separating gel. After completion of the electrophoresis, the gel was removed, stained with Coomassie Brilliant Blue R-250 for 30 min, destained in water, and then the proportion of each protein in the samples was analyzed by Image software 1.8.0.

#### 2.5.3. Moisture Distribution

Low-field nuclear magnetic resonance technology has the advantages of rapidity, nondestructiveness, and accurate state and flow characteristics of water molecules in MC powder through relaxation time separation and can directly reflect the state of water distribution and dynamic changes [20]. Approximately 1 g of MC powder was wrapped in plastic film and transferred into a sample tube for the measurement of moisture distribution using the low-field nuclear magnetic resonance instrument (NMI20-060V-I, Suzhou Numa Analytical Instruments Co., Ltd., Suzhou, China). The Carr–Purcell–Meiboom–Gill (CPMG) sequence parameters were set as follows: echo time, 0.2 ms; RF delay, 0.33 ms; waiting time, 800 ms; front gear, 3; and 8 accumulations [23].

#### 2.5.4. Fluorescence Spectroscopy

A protein sample was diluted with 1 × PBS buffer solution to the final concentration of 0.2 mg/mL. Intrinsic fluorescence spectrum was detected at 25 °C with the following parameters: excitation at 290 nm, emission scanning from 300 to 500 nm, slit width of 5.0 nm, and a scan rate of 600 nm/min. The main principle of protein intrinsic fluorescence is to detect the fluorescence of intrinsic amino acids (tryptophan, tyrosine, and phenylalanine), reflecting the tertiary structure of proteins.

Protein hydrophobicity was also examined using an anilino-naphthalene-8-sulfonic acid (ANS) probe. Protein samples were serially diluted to concentrations between 0.01 and 1 mg/mL in buffer. In each case, 10 μL of ANS solution (8.0 mM) was added to 1 mL of protein solution and allowed to stand in the dark for 5 min. Afterwards, fluorescence spectra were recorded with the excitation wavelength set at 380 nm, and the emission was scanned between 390 and 650 nm (slit width = 5.0 nm). The binding of ANS to the hydrophobic protein leads to a marked increase in fluorescence. The hydrophobicity index S_0_ was calculated from the initial slope of relative fluorescence intensity as a function of protein concentration [24].

### 2.6. Dispersibility and Stability Measurements

The dispersion stability of the powder samples was evaluated by monitoring time-dependent changes in particle size distribution using a high-performance Zetasizer Nano Series nanoparticle size and zeta potential analyzer (Model Nano-ZS3600, Malvern, UK). Prior to measurement, 0.5 g of powder was evenly dispersed in 50 mL of deionized water through magnetic stirring to the initially dispersed sample and then diluted 100 times with deionized water immediately before measurement [25]. All measurements were performed at a controlled temperature (25.0 ± 0.1 °C) at 2, 4, 6, 12, and 24 h.

After stirring the MC solution for 6 h, samples were transferred to a stability analysis vial to measure stability using Turbiscan LAB (Formulation, Toulouse, France) by multiple light scattering. Stability measurement was conducted at 25 °C for 18 h with scans every 10 min, and photographs of freshly prepared and stored samples were tanked for visual comparison.

### 2.7. Solubility Measurements

About 1 g (m_0_) of powder was dissolved in 50 mL of deionized water and stirred at room temperature using a magnetic stirrer for 0.5, 1, 1.5, 2, 4, 6, 8, and 16 h. The sample was then centrifuged at 3000× *g* for 10 min; then, the precipitate was collected in an oven and dried to a constant weight. The weight of the pellet (m_p_)was measured, and the solubility of the powder was calculated as follows [26].

Solubility (%) = (1 − (m_p_/m_0_)) × 100%

Here, m_0_ was the total weight of the powder; m_p_ was the weight of the undissolved pellet of MC solution after being dissolved and centrifuged.

### 2.8. Statistical Analysis

All measurements were performed in triplicate, and the results are expressed as mean values ± standard deviation. Statistical analysis and graph generation were performed using Origin (2021) software. Analysis of variance with Duncan’s test was carried out to analyze the significant differences. *p* < 0.05 indicated significance.

## 3. Results and Discussion

### 3.1. Composition and Structure of MC Powder

#### 3.1.1. Calcium and Phosphorus Content

Table 1 showed the dynamic changes of calcium and phosphorous content in MC powder after ED treatment. With the increased ED treatment time (from 0 to 90 min), the content of calcium in MC powder gradually decreased from 25.55 ± 0.08 g/kg to 17.47 ± 0.05 g/kg, indicating that the ED treatment could remove both colloidal and soluble calcium. It was reported that calcium depletion could destruct CCP crosslink and improve solubility [27]. This explains why ED-treated powder exhibited a better redispersion property. However, phosphorus content in MC powder exhibited biphasic behavior [28]. When applying 10 min ED treatment, the phosphorus content significantly increased by 0.27 g/kg (from 14.83 ± 0.04 g/kg to 15.10 ± 0.05 g/kg, *p* < 0.05), which possibly contributed to the release of phosphate due to the early stage of CCP dissociation, while with the 90 min ED treatment, the phosphorus content gradually decreased by 3.22 g/kg (to 11.88 ± 0.55 g/kg) due to the electromigration of free phosphates [29]. Therefore, the Ca/P molar ratio changed from 1.72 to 1.47. With the ED process, more calcium was removed from MC, while after 30 min, the mineral removal was more balanced.

#### 3.1.2. SDS-PAGE

**Figure 1:**
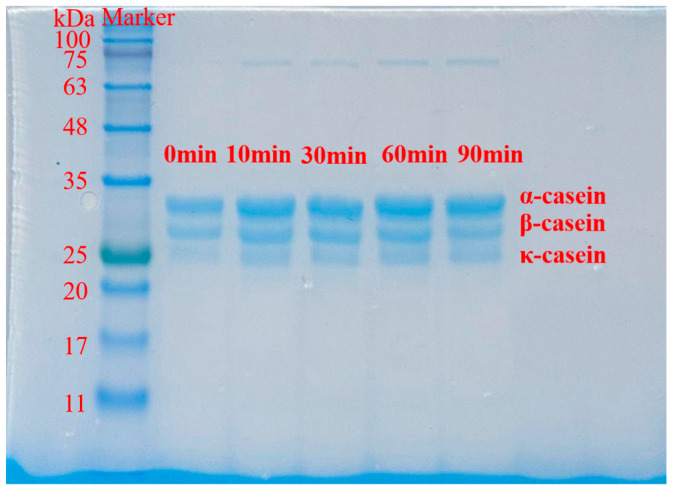
Gel electrophoresis of casein micellar solution was performed at different electrodialysis treatment times.

SDS-PAGE, effective for most proteins, mainly separates proteins according to their molecular weights [30]. The theoretical molecular weights of the main casein fractions are as follows: α_s1_-CN (23.6 kDa), α_s2_-CN (25.3 kDa), β-CN (24.0 kDa), and κ-CN (19.0 kDa) [31]. As illustrated in Figure 1, three protein bands were separated by the SDS-PAGE, namely α-CN, β-CN, and κ-CN (from top to bottom). The band intensities were quantified, and the relative amounts of the different casein fractions were not significantly affected by different ED treatment times, ranging from 54.1 to 55.8% for α-CN, 26.8 to 30.0% for β-CN, and 15.3 to 17.4% for κ-CN. Therefore, ED treatment did not alter the composition of the three casein fractions, indicating that the relative amounts of casein monomers did not account for the improved solubility. The enhanced MC solubility is more likely associated with ED-induced modifications in mineral content, particularly CCP.

#### 3.1.3. Moisture Distribution

**Figure 2:**
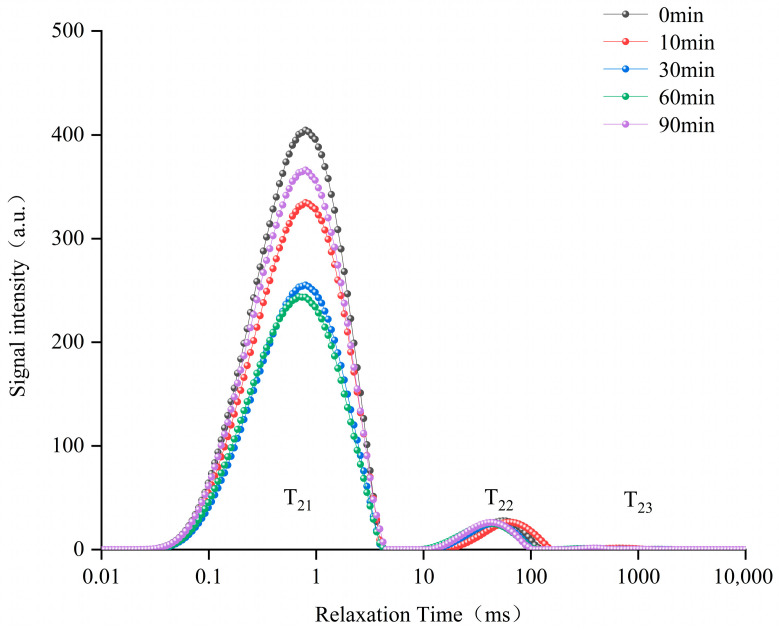
T_2_ relaxation times plots of MC powder at different electrodialysis treatment times. (T_21_ < 10 ms, T_22_ 10 ms ~ 100 ms, T_23_ > 100 ms).

The T_2_ relaxation time reflects the chemical environment, binding force, and mobility of hydrogen protons in samples [32]. T_2_ spectra of MC powder under different ED treatment durations exhibited distinct peaks (Figure 2), assigned as T_21_, T_22_, and T_23_, with corresponding peak areas A_21_, A_22_, and A_23_. Each relaxation time represents a specific water populations, while the peak area indicates its relative proportion [33].

The relative peak areas of these three water components in ED-treated samples are shown in Table 2. The results indicated that ED duration markedly influenced water distribution in MC samples, suggesting ED-induced water–protein interaction and changes in stromal structure.

Although the position of T_21_ (representing the tightly bound water of MC powder) was not changed during the ED treatment process, the relative area of T_21_ (A_21_) presented a typical biphasic response (Table 2 and Figure 2). After 60 min ED treatment, the content of bound water decreased from 96.70 ± 0.04% to 94.95 ± 0.06% but increased after 90 min. This rebound indicated that prolonged ED treatment (>60 min ED) induces casein molecule reorganization, exposing new hydrogen-bonding sites (e.g., polar groups of disrupted secondary structure) that enhance water-binding capacity. After 10 min ED treatment, the intermediate-mobility water (T_22_) moved to the right, suggesting that there was a new mobility increase on the surface, but T_22_ moved to the left after 90 min ED treatment, reflecting that water re-immobilization happened through the newly exposed internal sites [34]. The bulk-like water (T_23_) fluctuated irregularly due to the microstructural rearrangement. These results indicated that there was a three-phase change, including initial hydration disruption (0–30 min), internal changes (30–60 min), and significant molecular reorganization (>60 min). Therefore, 60 min was selected as the demarcation line between surface changes and substantial internal structure changes for MC.

#### 3.1.4. Fluorescence Spectroscopy

**Figure 3:**
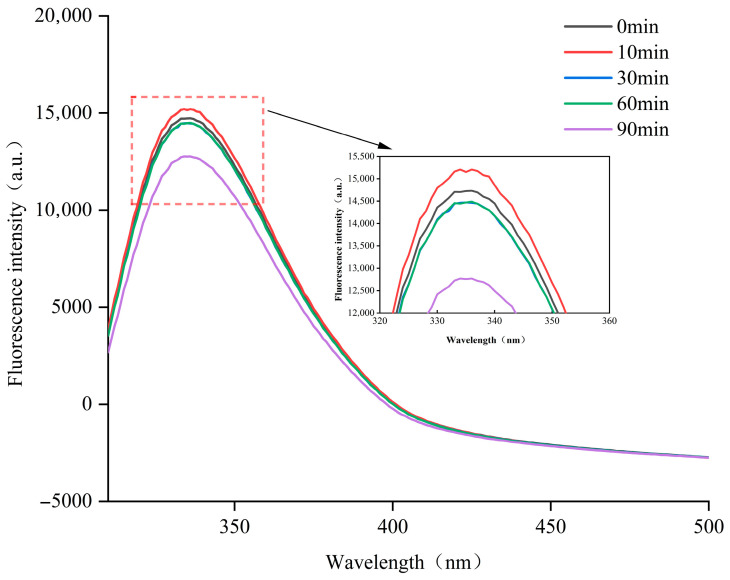
Effect of different electrodialysis treatment times on the intrinsic fluorescence intensity of MC.

Based on fluorescence spectroscopy, the effect of different ED treatments on MC was investigated. The intrinsic fluorescence of MC originated mainly from tryptophan (Trp) residues, and α_s1_-CN-contained 2 Trp residues (Trp 164 and Trp 199) and β-CN-contained 1 Trp residue (Trp 143), which were located at the hydrophobic domains and were highly sensitive to microenvironment changes. As shown in Figure 3, the fluorescence intensity increased initially and then decreased with an increase in ED treatment time. This phenomenon indicated that dynamic structural reorganization occurred in MC. Partial demineralization by short-term electrodialysis prompted initial MC dissociation, evidenced by a particle size peak after 10 min ED treatment, reflecting structural expansion. This expansion consequently enhanced microenvironmental flexibility, allowing greater rotational freedom for residues such as tryptophan. Long-term ED treatment further induced the rearrangement process that partially re-buried the Trp residues. Fluorescence changes indicated that ED can transiently modulate MC architecture by sequentially exposing and reorienting hydrophobic domains in response to calcium depletion and enhanced solubility.

**Figure 4:**
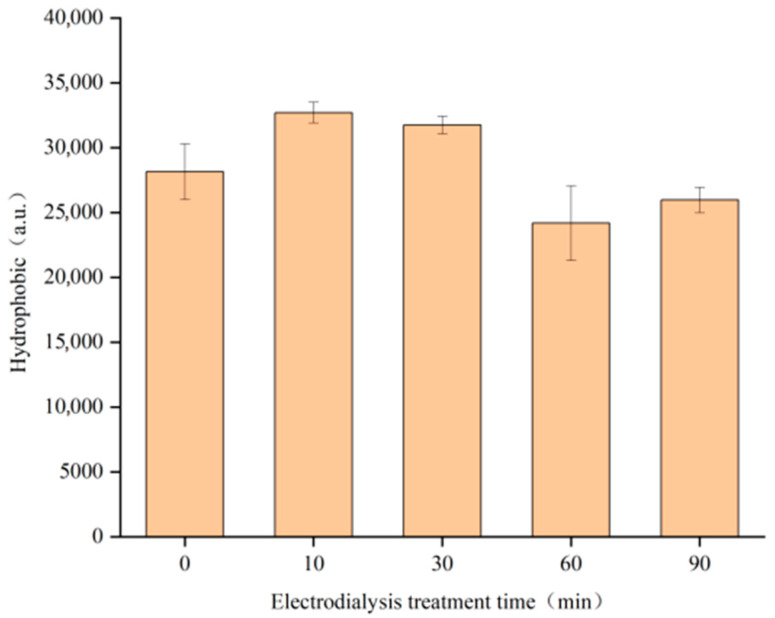
Effect of different electrodialysis treatment times on the hydrophobicity of casein surface.

Hydrophobic interactions govern protein conformation and protein–protein interactions [35]. Exposure of hydrophobic residues to the aqueous phase promotes water–protein interactions, stabilizing protein structure [30] as well as strongly influencing functional properties such as solubility, foaming capacity, and emulsification performance [36]. ANS has been reported to exhibit markedly enhanced fluorescence intensity when binding to hydrophobicity regions. By analyzing the relationship between protein concentration and fluorescence intensity, the initial slope (S_0_ = Hydrophobic) followed the trend of 10 min > 30 min > 0 min (untreated), while 60 min and 90 min treatments showed lower, similar S_0_ values (Figure 4). These results indicated that prolonged ED treatment (60–90 min) decreased hydrophobicity and improved solubility [37], possibly due to the rearrangement of the protein structure which re-embedded the hydrophobic region and generated new hydrophilic domains [38]. These results align with the well-established process of ED-modifying caseins, which could induce structural changes and improve functional properties.

### 3.2. Solubility of MC Powder

#### 3.2.1. Solubility Measurements

**Figure 5:**
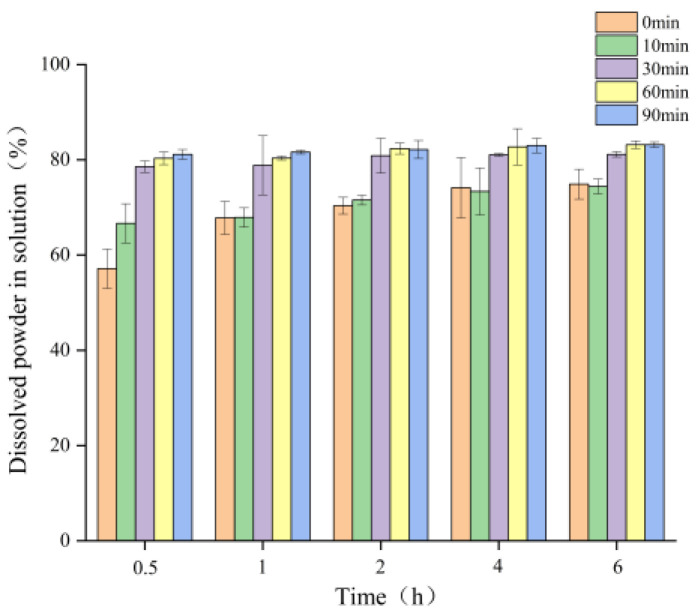
Solubility of micellar casein powder produced by freeze-drying after electrodialysis treatment.

Dissolution is the last step of powder rehydration, and the solubility of MC powder is assessed by measuring the solid content in the solution as a function of stirring time. Solid content of the untreated sample was <50% in the solution at 0.5 h in comparison with the ED-treated samples at 10, 30, 60, and 90 min (Figure 5). The untreated control exhibited lower solubility, while the ED-treated sample (10 min) also showed lower solubility relative to the untreated control after prolonged stirring. It may be related to the initial swelling of MC induced by short-term ED duration [39]. On the contrary, the ED-treated samples (30, 60, and 90 min) dissolved rapidly (≈80% solid content in the solution within 0.5 h). It may be attributed to the micellar calcium phosphate depletion induced by ED, leading to MC destabilization, and enhanced dissociation, consequently, improved solubility.

#### 3.2.2. Dispersibility Measurement

**Figure 6:**
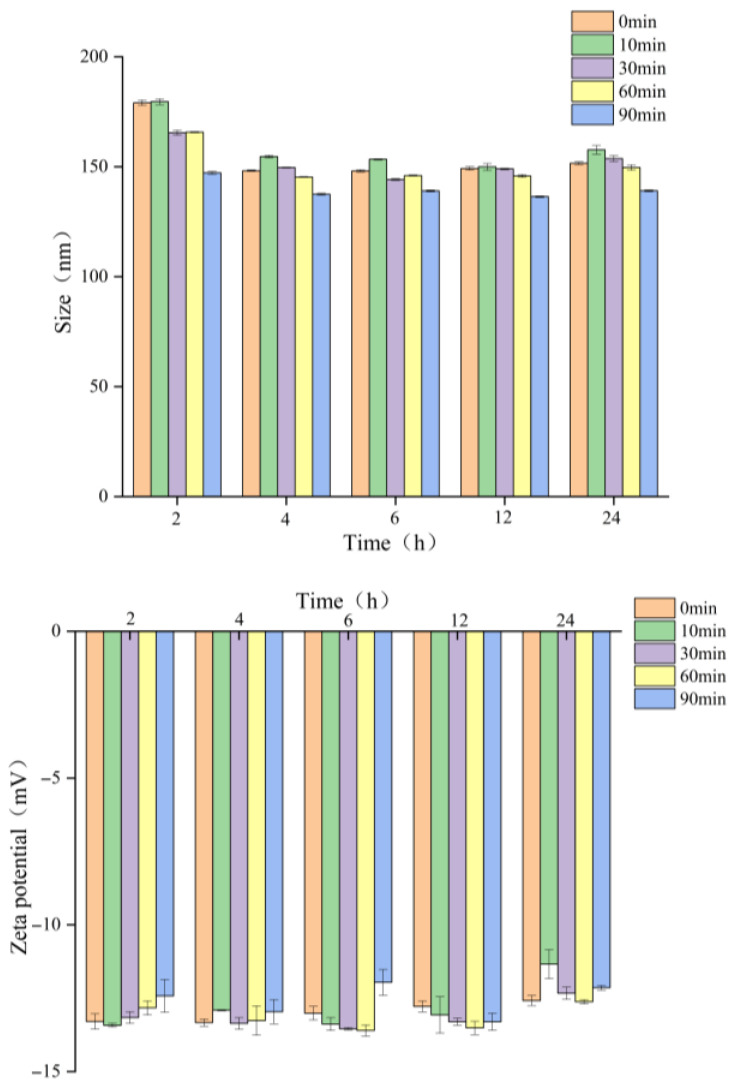
Changes in particle size and potential during dissolution of freeze-dried micellar casein powder after electrodialysis treatment.

**Figure 7:**
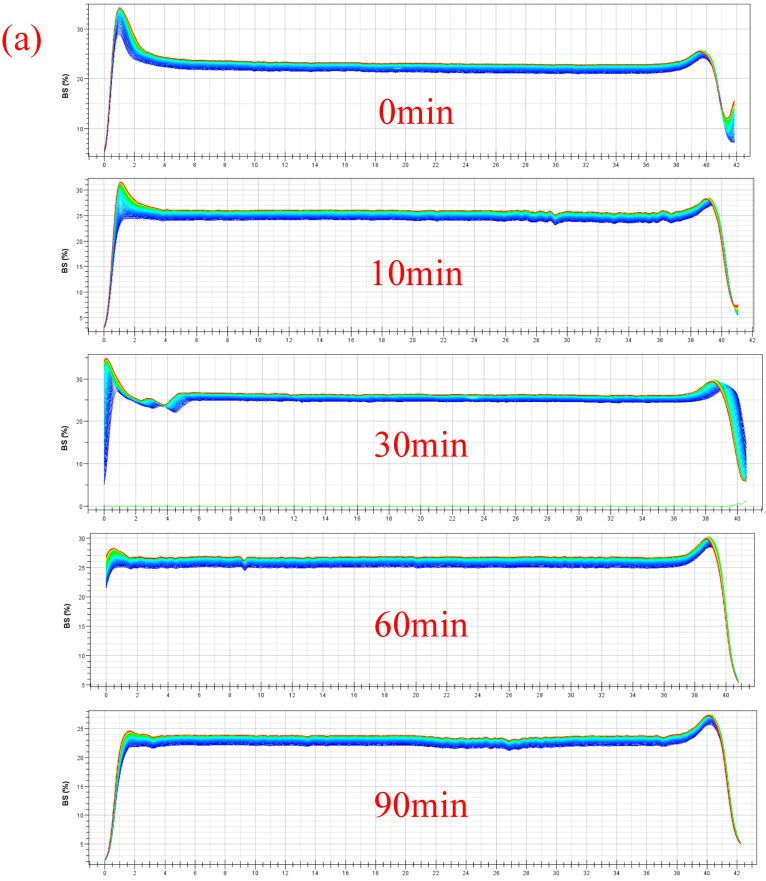

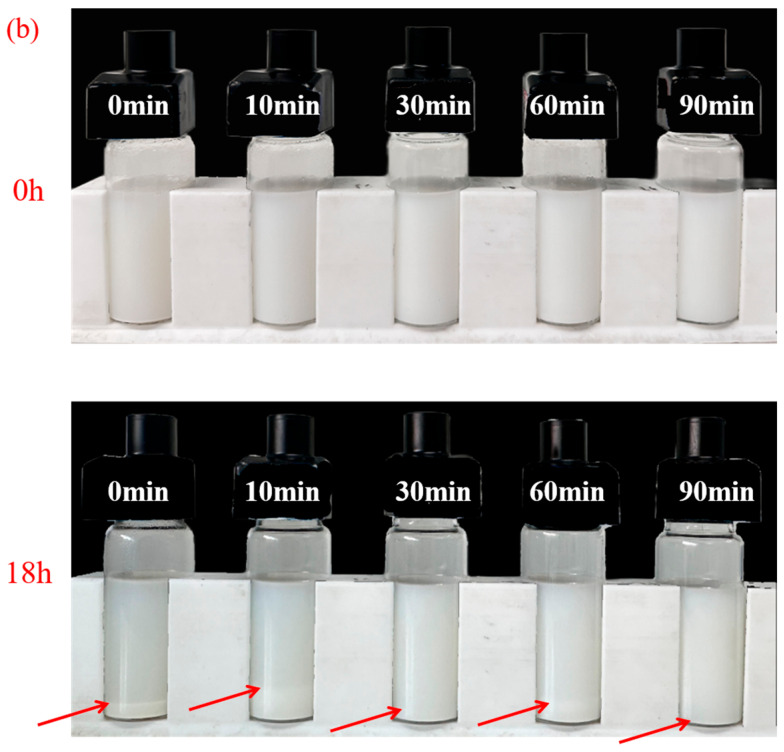
Stability of MC solutions. (**a**) Backscattering light diagram of electrodialysis-treated MC solutions; (**b**) Appearance of MC solutions at 0 h and 18 h. (0, 10, 30, 60, 90 min means the time of electrodialysis treatment).

Dispersibility of MC powder (ED treatment followed by FD) was assessed by measuring the particle size and zeta potential of reconstituted solutions (Figure 6 and Figure 7). Particle size of ED-treated samples decreased with the increased duration time. Nanoscale dimensions (<200 nm) of all samples were reached within 2 h after reconstitution, with the size of single MC ranging from 100 nm to 150 nm. After 4 h, the particle sizes of ED-treated samples followed the order 10 min > 0 min > 30 min > 60 min > 90 min. This trend suggests an initial disruption of micellar clusters during the early ED treatment stage (10 min), resulting in a transient size increase, followed by progressive dissociation into smaller subunits with extended treatment. In contrast, zeta potential exhibited irregular fluctuations throughout the ED process, which likely reflected dynamic surface charge modifications owing to mineral release and protein structural reorganization. These results indicate that ED treatment durations significantly affect the colloidal behavior of MC, while the optimal treatment duration (60–90 min) leads to the formation of a stable nano-dispersed system.

The stability of reconstituted MC powder was also investigated by light scattering for 18 h after 6 h dissolution. The backscattering profiles of different ED-treated samples exhibited variation in sedimentation as a function of sample height (bottom to top) [40]. Peak width was found to be correlated to the amount of sediment. The degree of sedimentation reached the highest value for the ED-treated sample (10 min), indicating the possible appearance of partial micelle dissociation and transient aggregation. With increasing ED-treated duration, sedimentation decreased and reached the lowest value after 90 min ED treatment (Figure 7). These results indicated that colloidal stability reached the optimal level after 90 min of ED treatment. Therefore, the trends in the above results can be explained as follows: the time-dependent structural reorganization of MC, which is initial disrupted (10 min ED treatment), lead to the formation of unstable intermediates, whereas completely dissociated MC (90 min ED treatment) lead to a uniformly dispersed system. These results were consistent with the smaller particle size obtained after prolonged ED duration.

## 4. Conclusions

This study investigated the effects of ED treatment on the rehydration of MC powder. These results showed that the increase in ED time significantly decreased calcium content (from 25.55 ± 0.08 g/kg to 17.47 ± 0.05 g/kg), suggesting that the CCP bridges, responsible for native micelles stabilization, are broken. In addition, ED-induced structural modifications are related to the improvement of the functional properties, i.e., greater intermediate-mobility water content, lower hydrophobicity, and, therefore, greater solubility and dispersion stability. However, short-duration ED treatment was insufficient to enhance solubility, while a marked improvement was observed only after prolonged treatment for at least 30 min. The solubility increased from approximately 50% (untreated MC) to about 80% (ED treated MC for 30–90 min) within the initial 30 min (0.5 h) of dissolution. Furthermore, optimal ED treatment duration (60–90 min) lead to the smaller particle size of casein micelle and the formation of a stable dispersed system. Thus, ED treatment greatly affects the MC structure and improves the rehydration properties of MC powder. Our results indicate that ED appears to be a good tool to tailor MC functionality. By enabling the production of MC powders with consistently superior solubility and stability, ED treatment can overcome long-standing challenges associated with the incorporation of casein ingredients into a wide range of food and beverage matrices.

## Figures and Tables

**Table 1:** Calcium and phosphorus content in MC powders produced with different ED treatment times.

Time (min)	Ca (g/kg)	*p* (g/kg)	Ca/P
0	25.55 ± 0.08	14.83 ± 0.04	1.72
10	25.35 ± 0.08	15.10 ± 0.05	1.68
30	24.18 ± 0.05	14.20 ± 0.05	1.70
60	21.93 ± 0.09	13.04 ± 0.05	1.68
90	17.47 ± 0.05	11.88 ± 0.05	1.47

**Table 2:** Proportion of three water peaks of MC powder with different electrodialysis treatment times.

Time	Bound Water (A_21_)	Immobile Water (A_22_)	Free Water (A_23_)
0 min	96.70 ± 0.04%	3.24 ± 0.00%	0.06 ± 0.04%
10 min	95.89 ± 0.13%	3.95 ± 0.00%	0.16 ± 0.05%
30 min	95.09 ± 0.03%	4.81 ± 0.00%	0.10 ± 0.03%
60 min	94.54 ± 0.06%	5.34 ± 0.00%	0.12 ± 0.09%
90 min	96.35 ± 0.03%	3.52 ± 0.00%	0.13 ± 0.02%

## Data Availability

The original contributions presented in this study are included in the article. Further inquiries can be directed to the corresponding authors.
